# Nursing care as perceived by trans persons: a phenomenological approach

**DOI:** 10.15649/cuidarte.3767

**Published:** 2024-09-01

**Authors:** Edgardo Álvarez-Muñoz, Sara Barrios-Casas

**Affiliations:** 1 Escuela de Enfermería, Universidad Santo Tomás, Temuco, Chile. E-mail: ealvarez14@santotomas.cl Universidad Santo Tomás Escuela de Enfermería Universidad Santo Tomás Temuco Chile ealvarez14@santotomas.cl; 2 Departamento de Enfermería, Universidad de La Frontera, Temuco, Chile. E-mail: sara.barrios@ufrontera.cl Universidad de La Frontera Departamento de Enfermería Universidad de La Frontera Temuco Chile sara.barrios@ufrontera.cl

**Keywords:** Nursing Care, Nursing, Gender Identity, Qualitative Research, Transgender Persons, Cuidado de Enfermería, Enfermería, Identidad de Género, Investigación Cualitativa, Personas Transgénero, Cuidados de Enfermagem, Enfermagem, Identidade de Gênero, Pesquisa Qualitativa, Pessoas Transgênero

## Abstract

**Introduction:**

Trans persons require particular and specific care, as well as equitable and egalitarian treatment corresponds to an ethical obligation for nurses. In health centers, the care relationship between nurses and trans patients remains unexplored in its entirety.

**Objective:**

To unveil the nursing care perceived by trans persons in Chilean health centers.

**Materials and Methods:**

Qualitative study, based on a phenomenological design according to Alfred Schutz. The data were collected through an in-depth interview conducted through Google Meet, then for the analysis of the data, the guidelines suggested by social phenomenology were followed. To safeguard the credibility, consistency and accuracy of the results, Lincoln and Guba's criteria of rigor were used.

**Results:**

The data were saturated with 9 participants from different regions of Chile. Four themes emerged from the research that describe the perception of trans persons in relation to the nursing care received: characteristics of humanized care; care in an ethical-legal perspective; facilitating factors for care and hindering factors for care.

**Discussion:**

Findings exposed in other studies were found, such as the need for gender training for nurses and the challenges and structural barriers faced by trans people; on the contrary, competent trans care practices and with respect to gender identity that differ from those referred to by other studies are highlighted.

**Conclusions:**

Nursing care perceived by trans persons is a complex phenomenon, influenced by various personal factors, experiences, feelings and emotions that shape the perception and quality of care.

## Resumen.

**Introducción:**

Las personas trans, requieren de cuidados particulares y específicos, así también, el trato equitativo e igualitario corresponde a una obligación ética para enfermería. En los centros de salud, la relación de cuidados entre enfermeros y pacientes trans permanece aún sin explorar en su totalidad.

**Objetivo:**

Develar el cuidado de enfermería percibido por personas trans en los centros de salud chilenos.

**Materiales y Métodos:**

Estudio cualitativo, con base a un diseño fenomenológico según Alfred Schutz. Los datos se recolectaron por una entrevista en profundidad realizada a través de Google Meet, posteriormente para el análisis de los datos se siguieron las orientaciones que sugiere la fenomenología social. Para resguardar la credibilidad, consistencia y precisión de los resultados, se utilizaron los criterios de rigor de Lincoln y Guba.

**Resultados:**

Los datos se saturaron con 9 participantes provenientes de diferentes regiones de Chile. De la investigación emergieron 4 temas que describen la percepción de las personas trans en relación con el cuidado de enfermería recibido: Características de un cuidado humanizado; Cuidado en perspectiva ético- legal; Factores facilitadores para el cuidado y Factores obstaculizadores para el cuidado.

**Discusión:**

Se encontraron hallazgos expuestos en otros estudios, como la necesidad de formación en género para enfermería y los desafíos y barreras estructurales que enfrentan las personas trans; por el contrario, se destacan prácticas de cuidado trans competentes y con respeto a la identidad de género que difieren respecto a lo referido por otros estudios.

**Conclusiones:**

El cuidado enfermero percibido por personas trans es un fenómeno complejo, influido por diversos factores personales, experiencias, sentimientos y emociones que configuran la percepción y calidad de este.

## Resumo.

**Introdução:**

As pessoas trans necessitam de cuidados particulares e específicos, da mesma forma, o tratamento equitativo e igualitário corresponde a uma obrigação ética da enfermagem. Nos centros de saúde, a relação de cuidado entre enfermeiros e pacientes trans permanece inexplorada em sua totalidade.

**Objetivo:**

Desvelar os cuidados de enfermagem percebidos pelas pessoas trans nos centros de saúde chilenos.

**Materiais e Métodos:**

Estudo qualitativo, baseado num desenho fenomenológico segundo Alfred Schutz. Os dados foram coletados por meio de entrevista em profundidade realizada por meio do Google Meet, posteriormente foram seguidas as diretrizes sugeridas pela fenomenologia social para análise dos dados. Para salvaguardar a credibilidade, consistência e precisão dos resultados foram utilizados os critérios de rigor de Lincoln e Guba.

**Resultados:**

Os dados foram saturados com 9 participantes de diferentes regiões do Chile. Da pesquisa emergiram 4 assuntos que descrevem a percepção das pessoas trans em relação ao cuidado de enfermagem recebido: Características do cuidado humanizado; cuidado na perspectiva ético-legal; fatores facilitadores do cuidado e Fatores dificultadores do cuidado.

**Discussão:**

Foram encontrados achados apresentados em outros estudos, como a necessidade de formação de gênero para a enfermagem e os desafios e barreiras estruturais enfrentadas pelas pessoas trans; pelo contrário, destacam-se práticas competentes de cuidado trans no que diz respeito à identidade de gênero que diferem do relatado por outros estudos. **Conclusões:** O cuidado de enfermagem percebido pelas pessoas trans é um fenômeno complexo, influenciado por diversos fatores pessoais, experiências, sentimentos e emoções que moldam a sua percepção e qualidade.

## Introduction

Globally, it is estimated that 25 million people identify as transgender and gender non-binary^1^, which has motivated states to implement strategies, policies and regulations to guarantee the right and protection of the gender identity of trans people (people whose gender identity is not congruent with their sex assigned at birth), promoting equality and respect for diversity^2^^, ^^4^. Despite these incipient anti-discrimination measures, even today the systemic victimization of trans people extends to high-, middle- and low-income countries, as well as many global cultural contexts^5^. Regardless of geographic location, transgender people exist within social contexts that stigmatize them^6^.

In most South American countries there is some type of anti-discrimination law, however, they do not have effective mechanisms to enforce these laws^7^. Particularly in the reality of Chile, different organizations that represent movements for the rights of sexual dissidence have criticized the State for not taking responsibility for preventing discrimination, despite the existence of incipient public interventions^8^, such as Law No. 21,120 of 2018, which recognizes the Right to Gender Identity^9^.

During 2012, the Chilean Ministry of Health disseminated Circular No. 21, which urges health establishments in the network to guarantee good treatment and respect for the dignity of trans people^10^. Despite this strategy, after 5 years, 95.00% of trans people declare that during care in health centers they have felt questioned about their gender identity^11^, probably due to lack of knowledge of their needs, which makes assistance difficult for these users^12^.

Trans people require particular and specific nursing care, not only from a non- pathologizing perspective, but also within the current legal framework, where the conception of nursing care based on a human rights approach understands that access to health and fair and equal treatment of all people is a basic social right^13^.

Around the world, trans people are exposed to human rights violations, such as social and employment discrimination, criminalization, pathologization, and exposure to transphobic violence. For organizations that defend their rights, there is a relationship between these human rights violations and the contemporary Western medical model of transsexuality, which psychopathologizes gender expressions and identities, which differ from social expectations related to the sex assigned at birth^14^.

The trans population is especially vulnerable to health disparities and inequities compared to cisgender people (people whose gender identity is congruent with their sex assigned at birth), experiencing higher rates of mental health disorders, eating disorders, consumption tobacco and alcohol, sexually transmitted infections, as well as marginalization, stigma and discrimination, are greater in this population^15^^, ^^16^.

From an ethical perspective on care, both the International Council of Nurses and the College of Nurses of Chile propose in their respective codes of ethics that nursing professionals promote environments in which human rights, values, the customs, religious and spiritual beliefs of people, families and communities^17^^, ^^18^.

Consequently, with this ethical perspective, nursing professionals are presented as highly relevant healthcare entities. They are responsible for providing direct care in health centers, can make decisions about the planning, execution and administration of this care and are committed to the provision of comprehensive care. In addition to playing a fundamental role in the fight for equity and justice in health care, as well as in reducing health disparities^15^. They must also protect privacy and communicate using patients' gender pronouns to provide trans-competent care; and from a clinical perspective, they must also have adequate knowledge about the health needs of these patients^19^.

Alfred Schutz's social phenomenology and his theory of social action explore how people experience and understand reality from their own perspectives. This approach conceptualizes all action as motivated behavior, dividing these motivations into two categories: “reasons to” and “reasons why.” “Reasons for” represent motivations related to the future, explaining the purpose behind a specific action, while “reasons why” refer to past experiences that influence the individual's behavior^20^. At the same time, for Schutz, the configuration of the subject is determined by intersubjectivity, which constitutes an essential characteristic of the social world. The “here” is defined because a “there” is recognized, where the other is. The subject can perceive reality by putting himself in the place of the other^21^.

Schutz's social phenomenology is useful for interpreting the meanings of the motivations and expectations shared by trans people. Thus, from this phenomenological perspective, nursing care is revealed as an experience between nurses and patients, transcending the limits of systems and levels of care.

These health systems strive to provide quality care in different aspects, such as direct care (health status), indirect care (resources), perceived care (individual and family perception) and demonstrated care (indicators), which are measured through indicators such as user satisfaction. However, a decrease is observed in the perception of the relationship between the health professional and the user, as well as in the understanding and appreciation of humanized care. This care is generally described with terms such as dignified care, humanization as comprehensive care, personalization of care and empathy^22^.

According to Ramos and Ceballos^23^, the act of caring in nursing establishes a nurse-user relationship, creating affection, concern and responsibilities between both. In this sense, for the authors, professional care becomes synonymous with humanized care. From a philosophical perspective, humanized care in nursing implies an ethical commitment to the dignity and uniqueness of the human being, as well as a process of continuous self-knowledge. Furthermore, it requires constant reflection on the problems of human interaction, addressing them from an ethical, social and political point of view, which underlines the importance of a comprehensive and holistic approach in professional practice.

In this context, the need arises to generate evidence that allows us to understand and develop relevant nursing care for this population group, to guarantee holistic and respectful care, with their unique needs and experiences. Therefore, the objective of the study is to reveal the nursing care perceived by trans people in Chilean health centers.

## Materials and Methods

The research corresponds to a qualitative study based on the phenomenological methodological design from the social perspective of Alfred Schutz, which is based on understanding the action of the subjects in the social world, taking as reference the intersubjective relationships inscribed in their daily experiences^24^^, ^^25^. The research was carried out under the conception of the constructivist paradigm.

The technique for data collection corresponded to an in-depth interview which was carried out by one of the researchers, master's student with experience in similar studies. The researcher did not have any type of relationship with the participants.

To recruit the sample, the key informant strategy and intentional sampling were used, through the coordinator of an Office of Diversity and Gender-Gender Dissidence of a municipality in Chile, who provided names of possible participants, who were contacted via telephone or email. Only 4 potential participants refused to participate in the study, citing disinterest or discomfort with the topic.

The inclusion criteria were Self-identify as a trans or non-binary person; Being over 18 years; Have experiences receiving nursing care in any health center and level of health care in Chile (primary or secondary). Those who were excluded were: who were receiving psychological/psychiatric treatment for any disabling mental health condition and who voluntarily stated that they did not want to participate in the research.

The interview was carried out between the months of August and October 2023. Only 2 participants requested to carry out the interview in person, the rest were carried out through the Google Meet platform, therefore, given the nature of the virtual platforms, it was not possible field notes were used.

The opening question for the interview was: “Could you tell me about your experiences in relation to the health system where you have been treated”, followed by other guiding questions such as: “Could you tell me about any experience or memory that has been significant for you?” “Any nurse who has treated you?” or “What aspects of nursing care do you consider most relevant or significant in your experience as a trans person in the health system?”

The interview was administered in a single session, recorded in audio format and then manually transcribed into text in Microsoft Word. To protect anonymity, participants used a code name chosen by them. The average duration of the interviews was 35 minutes. The interviews carried out were analyzed following the guidelines used by other socio-phenomenological researchers^25^^, ^^27^. These guidelines included several stages:

1. Thorough and repeated readings of each complete interview, with the initial objective of capturing the global meaning of the experience lived by the participants^25^^, ^^27^.
2. Identification and grouping of significant aspects extracted from the interviews to form specific categories that reflect the convergence of contents^25^^, ^^27^.
3. Rereading of the transcripts to identify specific categories and phrases that express relevant aspects of the participants' understanding and experience of reality, focusing on their experiences as trans patients receiving care in the health system^25^^, ^^27^.
4. Identification of categories that cover the acts of the participants and the interpretation of the meanings of these acts in the social context^25^^, ^^27^.

The 2 researchers of the study participated in each of the stages (a student and an academic from a master's program), subsequently the results were triangulated with 2 external researchers who were experts in qualitative research.

The content analysis began when theoretical data saturation was achieved according to Morse's guidelines^28^, obtaining a sample of 9 participants.

To safeguard the credibility, consistency and precision of the results, the rigor criteria proposed by Lincoln and Guba were used; 1. Credibility: a verbatim transcription of the interviews was carried out and used a phenomenological-constructivist approach, which allows a deep and contextualized understanding of the participants' experiences; 2. Transferability: the sample inclusion and exclusion criteria are described , as well as the recruitment strategies used, allowing other researchers to evaluate the applicability of your findings to similar contexts; 3. Dependency: a rigorous and transparent sample selection strategy was used, subsequently the data were analyzed based on the phenomenological analysis model; 4. Confirmability : the confidentiality and anonymity of the participants was guaranteed, and 4 researchers participated in the final validation of themes and subthemes. The results were subsequently returned to some participants, who evaluated and verified that the results reflected their experience, thus giving validity to the themes and subthemes that emerged^29^^, ^^30^.

The content collected from the interviews was anonymized and stored in Mendeley Data^31^.

The research was carried out respecting the 7 ethical requirements of Ezekiel Emanuel and had the approval of the university Scientific Ethics Committee, as stated in Minutes N°129_23, Folio N°109_23. All interviewees signed an informed consent to participate.

## Results

The sample was saturated with 9 participants with an average of 31 years of age. The 55.50% of the participants (5 people) declared themselves transfeminine, 33.30% (3 people) declared themselves transmasculine and only 11.20% (1 person) identified as non-binary gender. The sociodemographic characteristics are detailed in Table 1.

**Table 1:** Sociodemographic characterization of the participants

Key Name	Age	Gender Identity	Educational Level	Profession u Trade	Civil Status	Region
Juan	31	Trans Masculine	Complete University	Computer Civil Engineer	Single	La Araucanía
Lucas	22	Trans Masculine	Incomplete University	Tourism Engineering Student	Relationship	La Araucanía
Maria Paz	40	Trans Feminine	Incomplete University	Stylist	Relationship	La Araucanía
Esteban	29	Trans Masculine	Complete University	Social worker	Single	La Araucanía
Maria	33	Trans Feminine	Incomplete University	Event Producer	Relationship	Metropolitan
Abigail	36	Trans Feminine	Incomplete Doctoral Studies	Language and Literature Teacher	Single woman	Valparaiso
Yona	26	Non-Binary	Incomplete University	Does not declare	Single	Valparaiso
Romina	26	Trans Feminine	Full Half	Tattoo artist	Relationship	Bío-Bío
Sofia	34	Trans Feminine	Complete Master's Studies	Industrial designer	Relationship	Bío-Bío

Four themes of analysis emerged from the research: Characteristics of humanized care; Care from an ethical-legal perspective; Facilitating factors for care and Hindering factors for care. The themes and subthemes that emerged are detailed in Table 2.

**Table 2:** Themes and subthemes emerged from the study

Topics	Subtopics
Characteristics of humanized care	Provide care respecting human dignity Human quality in care
Care from an ethical-legal perspective Facilitating factors for care Obstacle factors for care	Respect trans identity Generational factor in nursing professionals Structural factors of health centers Bureaucratic factors of the health system Negative attitudes of professionals Nursing gender training needs

### Characteristics of humanized care

The first theme highlights how nursing combines technical competence with empathy and respect, safeguarding the dignity of the patient. This humanized care is understood through the "reasons for" of professionals, aimed at providing dignified and warm treatment. The experiences of the participants highlight actions that promote equal and comforting treatment, especially valued by trans people, who highlight the kindness and respect of the nurses. In this topic 2 subtopics were established:

### Provide care with human quality and respecting dignity

Under the perspective of social phenomenology according to Schutz, a relationship can be established between the care offered by nursing professionals and the "reasons for" that explain the purpose behind their actions.

The care delivered by nursing professionals acquires meaning through the lived experiences of the study participants. These participants highlight that nurses provide dignified care, reflected in concrete actions that safeguard human dignity and promote equal treatment. The importance of nurses' intrinsic qualities, such as empathy and compassion, is recognized, which have a profound impact on the perception of humanized, warm and comforting care.

In the integration of technical competence and human sensitivity, the act of caring becomes a deeply human experience, where the dignity of the patient drives each interaction. Through these interactions, participants interpret and give meaning to their experience of receiving nursing care in health centers.

*[I have never felt discrimination, to that point, they have always treated me well (..) they have been friendly, I have had good experiences] (Juan)*

*[particularly with male and female nurses I have had good experiences, they have treated me well and I have felt very good, because I believe that the equal treatment has been closer, I have felt as if in some way welcomed and respected in the pronouns] (Abigail)*

### Human quality in care

Trans people highlight the personality qualities of nursing professionals; these positive characteristics influence the delivery of care perceived as humanized, kind, comfortable and pleasant.

*[I don't remember the joke that a nurse once made to me, but it was something very simple that relaxed me... I think he was going to inject me with penicillin (...) he started to laugh, I don't remember, but I made up my mind and the treatment was ethical, he was respectful, he did not ask inappropriate questions like what has happened to me with other specialists] (Abigail)*

Abigail shares a specific experience that illustrates how she interprets and gives meaning to interactions with a nurse. From the perspective of social phenomenology, this care experienced in interaction with a nurse could have been motivated by past experiences that shaped the behavior and personality of the referred professional (“reasons because”).

### Care from an ethical-legal perspective

The second theme develops how respecting the identity of trans people is relevant as a sign of respect for their gender identity, based on current legislation and professional ethical regulations. This is reflected in care actions such as placing trans patients in rooms consistent with their gender identity, demonstrating respectful and dignified care. In this topic 1 subtopic was established:

### Respect trans identity

The study participants highlight the importance of using the social name and recognizing the physical changes associated with the transition process as indicators of respect for their gender identity. In this context, the care provided by nurses is valued positively when it promotes and respects the expression of their gender identity.

From Schutz's point of view, nurses, like any other individual, can understand reality by empathizing with the perspective of others, which makes it easier for common sense to identify others as like one's own self.

*[When I was admitted to the hospital, the nurses on duty sent me to a ward of all women, that is, they respected me in that they did not leave me with men] (Maria Paz)*

This experience of the participant reveals how trans people interpret and construct meaning around the actions of nursing professionals in relation to their gender identity. For Schutz, these interpretations are influenced by social expectations, past experiences, and concrete situations of interaction.

### Facilitating factors for care

The third theme reveals how the quality of nursing care is influenced by the age of professionals, highlighting how patients' experiences and perceptions are shaped by social interactions and experienced intersubjectivity. In this topic 1 subtopic was established:

### Generational factor in nursing professionals

For the participants, the quality of nursing care is influenced by the age of the professionals who provide it. In this sense, a difference is identified in the care received depending on the generation of the caregivers, with a greater appreciation of the care provided by younger nursing professionals compared to those of older age.

The difference in the care received depending on the age of the caregivers highlights how participants interpret and make sense of their interactions with nursing professionals through intersubjectivity. The differential assessment of care provided by younger professionals compared to older ones reflects how age becomes a factor that influences the perception of quality of care, framed in shared experiences within the social context of care medical.

[I *emphasize again that it is the current generations that are making the difference, because as I told you before, the bad experience I had was with an elderly woman, who was also a nurse] (Lucas) In Lucas's experience, an understanding of how trans people perceive and experience generational differences in nursing care can be provided.*

### Obstacle factors for care

The fourth theme describes how the factors that enable care in health centers include infrastructure, bureaucracy, professional attitudes, and gender training. In this way, physical and administrative barriers, along with discriminatory attitudes and lack of knowledge on gender issues, affect the quality of care perceived by trans patients. In this topic 4 subtopics were established:

### Structural factors of health centers

The study participants were able to identify a series of factors related to the infrastructure of health centers, with hygienic services being the physical spaces most recognized as places that make it difficult to feel that health centers are close and welcoming, which in turn has an impact in the quality of care perceived.

*[implement help with dissent, the bathrooms are “men's and women's bathrooms”, and they generally look at you ugly if you enter the women's bathroom if you are trans, because the men are right there next to the women's bathroom, so I think they lack spaces, they lack infrastructure] (Maria) [the vision remains binary at a structural level, just as man/woman and disabilities] (Romina)*

These experiences reveal how trans people interpret and attribute meaning to the structural factors of health centers from their own perspective, where the physical configuration of the spaces influences their sense of belonging and the perceived quality of care.

### Bureaucratic factors of the health system

There are a series of factors associated with bureaucracy in health centers, which constitute barriers to receiving optimal care and quality nursing care. These factors include administrative procedures, such as electronic records and legal name changes in health records, as well as long wait times to access specialized medical care. Some participants express that these obstacles could influence their decision to seek medical care in the future.

*[I went to complete the entire procedure and they told me that it was ready, but from what I understood, to each office that I go, I will have to enter my name, my identity from now on, so I prefer to avoid going to the offices for that very reason] (Lucas)*

*[at CESFAM when I wanted to enter due to the issue of harmonization and gender transition, I was on the waiting list for a year and they called me from the Van Buren Hospital to take the psychological test and that's as far as I got, then they never called me again (. .) apparently there is a big waiting list] (Abigail)*

Trans people's perception of bureaucratic factors in the healthcare system is understood as a shared construction of meaning, where administrative barriers influence their access to healthcare and their overall experience in healthcare facilities.

### Negative attitudes of professionals

Despite the existence ofa gender identity law and an anti-discrimination law in Chile, there is a smaller percentage of participants who highlight discriminatory attitudes on the part of nursing professionals, with the main critical event that can be identified being the lack of respect for the name. legal of the participants. These attitudes in many cases influence the continuity of care or the refusal to consult health centers in the future, as well as generate feelings of shame, sadness and frustration.

*[I was assisted by a lady who was a nurse, and I had to inject my testosterone and I asked her please if she could inject me, I showed her the prescription and everything, and she didn't believe me, she told me that the injection was too much (.. ) According to her, I needed less, even so, having the prescription that was from the endocrinologist] (Lucas)*

As Schutz suggests about the “reasons because,” the experiences of verbal discrimination experienced by trans people like Lucas may be motivated by experiences rooted in the past and the personality developed throughout the lives of nursing professionals.

### Nursing gender training needs

The study participants recognize that, although nursing professionals show a respectful approach in the care they provide, they identify a lack of knowledge on gender issues. This is considered essential to improve the quality of nursing care provided to this population.

*[there are nurses who don't know much about trans boys or trans girls, so they should educate themselves a little more or, I don't know, there should be some gender education that can be implemented in clinics or hospitals] (Lucas)*

These demands reflect the importance that trans people attribute to receiving nursing care that is sensitive to their gender identities and individual experiences. Lucas, by expressing this need, is sharing his experience and his vision of the world, where a lack of understanding about the experiences of trans people in the context of health care is perceived as a barrier to receiving quality care.

In the context of social phenomenology according to Schutz, the relationship between the "reasons because" and the lack of knowledge on gender issues on the part of nursing professionals can be understood due to their personal and cultural background, insufficient academic training and training, the lack of exposure and experience in diverse environments, as well as prejudices and stereotypes ingrained in society.

## Discussion

The objective of this study was to reveal the nursing care perceived by trans people in Chilean health centers. In this sense, the majority of participants declare that they receive humanized care from nursing professionals, which was reflected in the subtheme Providing care that respects human dignity, the same as what was reported by two studies from the United States in which Trans people report having received compassionate, kind treatment that respects their pronouns; These characteristics were identified as the main actions of nurses, which make them feel comfortable during their care^32^^, ^^33^.

In contrast, when analyzing the participants' speeches, few findings were found in relation to what was reported in other investigations, in which trans people state that nurses do not present an empathetic attitude towards them, they provide prejudiced care regarding their health sexually, carry out discriminatory acts or carry out situations of psychological abuse^15^^, ^^34^^, ^^37^.

From an ethical perspective, when analyzing the participants' reports, it is deduced that nurses act in accordance with the principle of respecting human rights, values, customs and spiritual beliefs of people, as established in the Code of Ethics of the College of Nurses of Chile^18^. The results mostly reflect adherence to these ethical principles, which is encouraging and suggests a commitment to providing quality and respectful care to all people, including those with diverse gender identities.

The importance of respecting the social name and self-perceived gender identity is highlighted in the study's Respecting Trans Identity subtheme, which resonates with similar findings found by Núñez et al.^38^ in a hospital in Chile, where trans people receiving medical care expressed that the recognition of their social name in the registry, clinical history and during interactions with health personnel generates beneficial feelings and contributes to a more positive care experience.

These aforementioned findings are consistent with the principles established in the law that recognizes and protects the right to gender identity in Chile^9^. Along these lines, according to a study from Uganda, nursing professionals express their intention to offer care without prejudice, use language and terminology appropriate for trans people, such as pronouns, and create transgender-sensitive environments in health centers^37^. Likewise, Australian trans people state that one of the most valued aspects of nursing is their willingness to listen, learn and respect the person's name and pronouns, which they consider essential to feel supported^39^.

Although to a lesser extent, some of the participants express situations in which their identity is not respected, just like Von Vogelsang et al. ^40^ describes it in the experiences of trans people in Sweden.

It is interesting to highlight as a new finding of the study, the subtopic Generational factor of nursing professionals, where no similar results were found in the reviewed literature, only one Australian investigation mentioned that trans participants would assume that health students more young people may have greater awareness of their problems, however, the authors were unable to reflect this situation in their results^41^.

Along these same lines, Valenzuela-Valenzuela et al. ^42^ published a Chilean study carried out on trans people, where it was concluded that health professionals themselves belonging to certain groups (such as young people, women and migrants), present a greater general openness towards trans care issues.

In the results presented in the subtopic Structural factors of health centers, findings were found that are consistent with another study carried out on Peruvian trans men^34^, who describe hygienic services as critical and problematic spaces, which generate conflicts, as they are recognized as stereotypical places within the “man/woman” “ladies/gentlemen” binary.

At the ministerial level, different clinical guides, guidelines and regulations have been presented, aimed at improving the flow of care for trans people in the country's health facilities^43^^, ^^44^. Despite all these strategies, the study participants continue to perceive some barriers to effective care, which were grouped in the subtopic Bureaucratic factors of the health system, In this sense, other studies have described system problems as critical factors, such as electronic documentation, admission forms and confidentiality^45^, presenting an identity card that reflects the sex assigned at birth^34^, waiting and the cost of care^35^. All of these aforementioned factors can influence the decision to consult in new health centers in the future^32^^, ^^45^.

A finding that has become recurrent in the results of this study is the identification of gender training needs among nursing professionals. This issue has been highlighted significantly by the participants, who have expressed the need for nurses to receive more complete training on gender and sexual diversity issues, like what is evident in other Chilean studies, where trans people play a significant role, the lack of information regarding gender and sexual diversity issues on the part of professionals who work in health facilities^45^.

In relation to the above, Reisner et al. ^34^ showed that trans people report a general lack of awareness and information about transsexual identity and health needs among health personnel; Likewise, Sousa et al.^47^, established that training in gender is important for the training and qualification of professionals in their practices.

When looking at the disciplinary foundations of nursing, it becomes evident that gender transition not only involves physical changes but can also generate significant transformations in multiple aspects of a person's life. Afaf Meleis^48^ maintains that gender transition can trigger profound changes in identities, roles, relationships, skills and behavioral patterns, which is reflected in the health experiences shared by study participants. This understanding is fundamental to nursing practice, as it recognizes the complexity and breadth of the needs of people undergoing gender transition processes.

Social phenomenology offers a valuable contribution to understanding the role of nursing in interacting with trans people. This interaction implies a social relationship between two subjects: the caregiver and the cared for.

From Schutz's perspective^21^, nursing care for trans people, understood as a complex phenomenon, can be analyzed through the underlying motivations for care, as reported by the study participants. This involves exploring both the “reasons because” and the “reasons for” that justify nurses' actions from the perspective of trans people, as well as elaborating the contexts of meaning in which these interactions take place. Care is based on intersubjectivity, the body of knowledge and the biographical situation of the professional caregiver^25^.

## Conclusion

The study fulfilled its objective by revealing how trans people perceive nursing care in Chilean health centers. This care is a complex phenomenon, influenced by various personal factors, experiences, feelings and emotions that shape the perception and quality of the care received. These results reflect the complexity inherent in nursing science, which provides comprehensive care to people in all their dimensions: physical, emotional, social and spiritual. It is essential to recognize that nursing care relevant to the needs of trans people goes beyond just the technical aspects; Therefore, competent trans care is more valued and recognized.

From an ethical-legal perspective, it can be observed how legal regulations and public policies aimed at protecting the rights of trans people significantly impact the quality of care provided by nurses. Trans people can recognize in nursing actions such as respect for the social name of those who have not changed their sex and legal name, making the gender identity of individuals visible by treating them with their self-identified gender, and providing carefree of arbitrary discrimination and transphobic violence.

Given the, it is important that legislators continue to generate instances that allow the current laws to be improved, with the aim of maintaining the rights of trans and gender non-conforming people.

In this sense, nursing professionals can play an active role in guiding the regulations issued from the central level, as well as being participating entities to advise the country's legislators in this challenge.

This study has made it clear that factors related to the infrastructure of health centers and the administrative processes of access and opportunity to care significantly influence the health experiences of trans people, and, therefore, the care of Nursing. Therefore, it is crucial to overcome these barriers and transform health centers into more inclusive spaces for sexual dissidence.

Given the revealing results regarding the lack of training in gender issues among nurses, the individual responsibility of each professional to acquire knowledge in this area is highlighted. It is imperative that universities and higher education institutions incorporate topics related to gender and attention to the LGBTQ+ (Lesbian, Gay, Bisexual, Trans, Queer, +) population in their study plans. It is time for the training curriculum of future nursing professionals to provide an adequate response to the care of gender diversity with sensitivity and competence.

Finally, the research is presented as a precedent in the context of studies on care for sexual and gender dissidents in Chile, being a contribution to show how nursing can contribute to achieving greater social equity for a vulnerable population group.

Therefore, the invitation to continue researching the topic is left open and the concern is raised to form associations and scientific societies, specific to nursing care in sexual dissidence, to generate spaces for discussion and reflection to improve trans health care.

The characteristics of the sample are considered as a limitation, in which there is low heterogeneity both in the educational level and in the age of the participants, which could have biased part of the results, not addressing the experiences of older trans people or people with minors level of schooling. Therefore, the results presented must be interpreted with caution.
